# Testing effectiveness of the revised Cape Town modified early warning and SBAR systems: a pilot pragmatic parallel group randomised controlled trial

**DOI:** 10.1186/s13063-019-3916-0

**Published:** 2019-12-30

**Authors:** Una Kyriacos, Debora Burger, Sue Jordan

**Affiliations:** 1 Division of Nursing and Midwifery, Department of Health & Rehabilitation Sciences, Faculty of Health Sciences, University of Cape Town, Cape Town, South Africa; 2 School of Human and Health Sciences, Swansea University, Swansea, Wales, UK

**Keywords:** Early warning score, Decision making, Healthcare systems, Nurse training (MESH terms: early diagnosis, monitoring – physiologic/nursing, vital signs)

## Abstract

**Background:**

Nurses’ recognition of clinical deterioration is crucial for patient survival. Evidence for the effectiveness of modified early warning scores (MEWS) is derived from large observation studies in developed countries.

**Methods:**

We tested the effectiveness of the paper-based Cape Town (CT) MEWS vital signs observation chart and situation-background-assessment-recommendation (SBAR) communication guide. Outcomes were: proportion of appropriate responses to deterioration, differences in recording of clinical parameters and serious adverse events (SAEs) in intervention and control trial arms. Public teaching hospitals for adult patients in Cape Town were randomised to implementation of the CT MEWS/SBAR guide or usual care (observation chart without track-and-trigger information) for 31 days on general medical and surgical wards. Nurses in intervention wards received training, as they had no prior knowledge of early warning systems. Identification and reporting of patient deterioration in intervention and control wards were compared. In the intervention arm, 24 day-shift and 23 night-shift nurses received training. Clinical records were reviewed retrospectively at trial end. Only records of patients who had given signed consent were reviewed.

**Results:**

We recruited two of six CT general hospitals. We consented 363 patients and analysed 292 (80.4%) patient records (*n* = 150, 51.4% intervention, *n* = 142, 48.6% control arm). Assistance was summoned for fewer patients with abnormal vital signs in the intervention arm (2/45, 4.4% versus (vs) 11/81, 13.6%, OR 0.29 (0.06–1.39)), particularly low systolic blood pressure. There was a significant difference in recording between trial arms for parameters listed on the MEWS chart but omitted from the standard observations chart: oxygen saturation, level of consciousness, pallor/cyanosis, pain, sweating, wound oozing, pedal pulses, glucose concentration, haemoglobin concentration, and “looks unwell”. SBAR was used twice. There was no statistically significant difference in SAEs (5/150, 3.3% vs 3/143, 2.1% *P* = 0.72, OR 1.61 (0.38–6.86)).

**Conclusions:**

The revised CT MEWS observations chart improved recording of certain parameters, but did not improve nurses’ ability to identify early signs of clinical deterioration and to summon assistance. Recruitment of only two hospitals and exclusion of patients too ill to consent limits generalisation of results. Further work is needed on educational preparation for the CT MEWS/SBAR and its impact on nurses’ reporting behaviour.

**Trial registration:**

Pan African Clinical Trials Registry, PACTR201406000838118. Registered on 2 June 2014, www.pactr.org.

## Introduction

Nurses are responsible for monitoring patients for signs of clinical deterioration. These might be most effectively detected using bedside early warning scoring (EWS) systems and a standardised reporting system such as the situation-background-assessment-recommendation (SBAR) guide. In 2010 we conducted our first pragmatic, cluster randomised controlled trial (RCT) of the original, consensus-derived, Cape Town (CT) modified EWS (MEWS) observations chart in three intervention and three control adult surgical wards in a single research site [1–3]. At the time, electronic track-and-trigger MEWS and reporting systems were increasingly widely used in healthcare settings in developed countries [4–6], whereas pen-and-paper systems were slowly being implemented in developing countries [1, 7]. As in our trial, government sector hospital wards in low-income and middle-income countries in Africa used pen-and-paper graphic charts to plot temperature, blood pressure, heart and respiratory rates, without information on normal or expected values [1]. Paper charting of observations takes longer than electronic systems, increasing nursing workload [8] and risk of human error [9], particularly inaccuracy of calculations [10].

From our trial, we concluded that the original CT MEWS chart and training programme enhanced recording of respiratory rate and all parameters, and nurses’ knowledge, but not nurses’ responses to patients who triggered the MEWS reporting algorithm. At the conclusion of the trial we were required to withdraw the CT MEWS because this was an independent rather than a government-endorsed study. Nevertheless, we were inspired by the Royal College of Physicians’ National Early Warning Score (NEWS) [11] to revise the CT MEWS chart under the Creative Commons Attribution-No Derivatives 4.0 International Licence (Additional file 1), and to undertake a further RCT.

Changes in clinical parameters, such as pallor and reduced level of consciousness, and abnormal physiological vital signs herald patient deterioration, often 6–8 h before the onset of a serious adverse event (SAE) [12]. To help nurses recognise deterioration, physiological values are recorded on MEWS charts within partitioned coloured bands (cut points or thresholds) with corresponding colour-coded weighted trigger points (scores) (0, upper and lower 1–3) (Additional file 1). If nurses are not adequately trained in recognition of deterioration, absence of trigger points may restrict nurses’ capacity to summon clinical assistance when needed.

Educational programmes normally accompany implementation of EWS/MEWS [13]. A systematic review reported effectiveness of educational interventions (mainly simulation) in recognition and management of deteriorating patients, improved patient outcomes and organisational systems. The review identified 20 quantitative studies, 2 mixed methods studies and 1 qualitative study (total *n* = 23): 11 of 20 (55.0%) studies indicated a statistically significant improvement in knowledge, clinical performance, use of medical emergency teams (METs), frequency of observations, and patient outcomes [13]. Improvement in confidence in approaching senior staff for advice and working as a team were also reported [13].

A systematic review of 11 articles (10 studies) on the effect of EWS systems training reported a statistically significant improvement in either nurses’ knowledge or confidence or clinical performance [14]. All studies (but one) were conducted in countries with high human-development indices [14].

It is not surprising, therefore, that we were unable to locate reports of the introduction of EWS in government sector hospitals in developing countries. At the inception of this study, the public healthcare sector in South Africa had commenced transitioning to a National Health Insurance (NHI) system [15]. The outcome is a health financing system designed to pool funds and actively purchase services to provide access to high quality, affordable personal health services for all South Africans, irrespective of socioeconomic status. The final phase is scheduled for 2022. A pen-and-paper recording system will make it difficult to achieve objective evaluation of the quality of nursing care: like all evaluations, this will require data capture, storage and analysis to guide a standardised approach to clinical care [16].

In the present study, we aimed to test the effectiveness of the revised instrument (Additional file 1) and a locally validated MEWS-linked SBAR guide (DB) (Additional file 2) on hospital nurses’ performance in early identification and paper reporting of clinical and physiological deterioration in adult patients in public sector teaching hospitals in Cape Town, South Africa. As in our first RCT of the original CT MEWS chart, the second RCT of the revised MEWS was conducted *de novo* without the nurses having any prior knowledge of EWS for monitoring patients’ vital signs. To our knowledge this is the first multi-site RCT on the effectiveness of a MEWS vital signs observations chart for early recognition and response to signs of deterioration [3].

## Methods

### Design

This was a pilot, pragmatic, parallel-group RCT with two arms (intervention versus usual care). Pragmatic clinical trials (PCTs) are randomised trials designed to compare the effectiveness of interventions in real-world settings [17] for the primary purpose of informing decision-makers of the balance of benefits, burdens and risks [16]. Here, hospitals were the unit of randomization, that is, random allocation of all participating general medical and surgical wards in the hospital to the same trial arm. There was contamination bias in our previous single-site trial due to nurses working across wards [3].

### Ethical considerations

We regarded our study as being of minimal risk, limited to “interventions already used in routine clinical care” ([18]:486). The local University of Cate Town (UCT) Clinical Research Centre (CRC) was the trial sponsor. The trial protocol received ethical approval from the Local Human Research Ethics Committee (HREC 337/2014), the local Provincial Department of Health (2014RP069), and the National Department of Health (DOH-27-0614-4779). They determined that patients would be recruited by informed, written consent for use of the revised MEWS chart and record review (after the intervention). In the earlier trial (HREC 192/2009) [3], individual patient consent for these processes had been deemed unnecessary.

In this second RCT, recruitment by individual patient consent soon proved impractical and hindered the delivery of the trial. We then submitted a revised protocol, requesting a waiver of individual written consent. Subsequent revised HREC approval (Ref. 825/2014) required written permission from gatekeepers for such a waiver and hospital notices to this effect in A3 size. This was not acceptable to some hospitals. For example, one academic tertiary hospital declined participation, stating that the display of A3 posters in research wards, replacing individual patient consent, would breach their value framework and might result in family or staff unhappiness or public media inquiries or complaints. We then used the earlier approval (HREC 337/2014), requiring individual patient consent, without posters.

### Setting

All six acute, government-sector hospitals for adult patients in the Western Cape, South Africa were matched for level of acuity and patient numbers (three pairs) within a predetermined distance range (124 km) from UCT, and approached (recruitment success is described under “Results”). Previously, in our first single-site study [3], we observed that some staff worked across several wards in their hospitals, often as relief or bank nurses. Therefore, to limit contamination in this RCT, each hospital was either an intervention or a control site. Before the study we obtained, with permission, encrypted, fully anonymised data from the Western Cape provincial Department of Health database for February 2014 (admission numbers, death rate, length of stay). We compared data to ensure that we were comparing wards and hospitals with similar reporting levels for these parameters. The trial was conducted on general medical and surgical wards. Specialist units such as intensive care were outside the scope of the study. Data were obtained by retrospective review of patient records for both trial arms, following the educational intervention and trial use of the MEWS chart and SBAR guide in the intervention arm.

### Screening and randomisation

The Swansea Clinical Trials Unit randomly allocated hospitals electronically to either the intervention or control trial arm before commencement of the study. As hospitals became available they were randomised in pairs (pre-matched by acuity and type, as above). There were no statistically significant differences in mortality rates between the hospitals paired for randomisation. General medical and surgical wards were matched, resulting in the selection of two matched surgical and one medical ward in each hospital allocated to their hospital’s trial arm. The process of consenting nurses to training or no training in the respective trial arms (hospitals) precluded blinding.

### Outcomes

The primary outcome was nurses’ reporting behaviour, defined as proportion of responses to early signs of clinical and physiological deterioration judged as appropriate, using the CT MEWS criteria for critical deterioration labelled as “red MEWS” and scoring a 3. Secondary outcomes were the differences between intervention and control arms in (1) number of patients with recordings in the order that these appear on the MEWS chart (respiratory rate, oxygen saturation, temperature, heart rate, systolic blood pressure (BP), level of consciousness, urine output), (2) recordings of all seven vital signs, (3) nurses’ responses to MEWS, and (4) the proportion of patients who developed serious adverse events (SAEs) [19]. We recorded patients’ demographic and clinical characteristics, and prescription of opiates, sedatives, diuretics and anti-hypertensives received on the day of vital sign recordings.

## Participants

### Patient records for retrospective review

We aimed to evaluate effectiveness of the revised MEWS chart by reviewing records of consented patients aged ≥ 18 years, admitted in August 2014 to the study wards, with the following exclusions: noted as “Not for Resuscitation”, transferred out of the ward to another department within 12 h following admission, or pregnancy. Clinical records of consented patients were excluded if:

1. Unavailable: in the medico-legal department (for example after death); in the x-ray department; removed for teaching purposes or transcription; in active use in association with re-admission of the patient; or
2. Incomplete: without a vital signs chart (in the intervention wards, the MEWS chart) or the nursing record/progress notes; or
3. Missing for unknown reasons.

Patients with multiple drug-resistant pulmonary tuberculosis were included only after data collectors were fitted with the correct masks.

#### Sample size for record review

Our original sample size calculation was based on our previous RCT of the CT MEWS (Trial registration PACTR201309000626545) [3], which detected a difference of 9% between the two arms in the numbers of patients whose abnormal vital signs triggered calls for assistance (7/53, 13.2% vs 2/52, 3.8%). An effective sample of 300 records (150 from each arm) would have been sufficient to detect a difference of 9% (4–13% increase) between arms in summoning appropriate assistance with 80% power and 5% significance. This calculation took no account of clustering [20, 21]. The intra-cluster correlation coefficient for numbers of patients for whom responses were triggered appropriately was calculated to be 0.02 (based on comparison of variance between and within clusters) [21]. With 12 wards potentially available and 51 patients in each ward, the design effect is 2.0. Therefore, to give an effective sample size of 300, 612 patients had to be recruited, 51 in each of 12 wards [21].

### Ward nurses’ training and implementation of MEWS and SBAR

All nurses in full-time employment in the three intervention wards (31 nurses on day floor duty plus 17 on night duty, *n* = 48), were eligible for participation. There were 26 beds in each of two wards, and 30 in the third ward (*n* = 82 beds). The 31 “day staff” across the three wards comprised 9, 10 and 12 nurses, respectively, split into two teams, working on different days of the week. External agency nurses were hired (4 for day duty and up to 10 for night duty depending on patient load) to cover staffing deficits. Agency nurses on day duty did not receive training, even though they would be using the research instruments, because they were allocated to different hospitals from day to day.

The eight hospital nurse managers, who did not do floor duty, did not participate in the training sessions. We were given a training schedule allowing 24/31 (77.4%) ward nurses on day floor duty in the three intervention wards to attend on-site training; 3 clinical nurse ward operational managers and 4 ward nurses were excluded from the training schedule. No reasons were given for their exclusion. Six groups of 3–5 informed, consenting nurses per session were trained (*n* = 24: 8 Registered Professional Nurses (RPNs), 3 RPNs on a compulsory 2-year community service post-training programme, 6 Enrolled (Staff) Nurses and 7 Nursing Auxiliaries).

Informed, consented nurses on night duty (*n* = 23) from the three intervention wards were trained on site in the evenings over the same 6 days. Of these, 17 were in full-time employment and 6 were external agency nurses consistently stationed on night duty in the intervention wards.

#### Intervention (1): training programme for intervention wards

An interactive 8-h classroom training programme on the CT Mews chart and MEWS-linked SBAR communication guide was reduced by hospital managers to 2 h face-to-face teaching. Day duty nurses were trained over 6 days in July 2014, a month before testing the instruments (1–31 August 2014). The training programme (by UK, DB) consisted of a MEWS and SBAR pocket guide, revision of basic anatomy and physiology, followed by recording vital signs and clinical data on blank MEWS charts from hypothetical patient scenarios (DB). Data on MEWS charts were then transcribed onto SBAR forms and used for role play for calling the doctor. All nurses were offered additional training for up to 8 h but no-one accepted. Overall, 24/31 (77.4%) day staff and all the night staff received training. In the control arm, nurses were informed and consented to record review, but received no training. Nurses in both trial arms gave written informed consent to participate. We would have excluded those unwilling to give signed, informed consent, but this was unnecessary, as all those approached gave written consent to participate.

#### Intervention (2): 31-day use of MEWS/SBAR on intervention wards

In both trial arms, we presented the study to senior nurses, doctors and hospital managers. Nurses in both trial arms received a written and verbal explanation in English, the medium of instruction in local nursing educational institutions. In the intervention wards, nurses, managers and doctors agreed to use the CT MEWS chart and SBAR guide for monitoring patients’ vital signs for 1 month. In control wards, nurses used the usual observation chart (Additional file 3), and, if a patient’s condition deteriorated, they followed their usual procedure for calling for help. When patients were discharged, researchers reviewed patients’ charts for the quality of vital signs recordings in both trial arms.

Neither the MEWS chart nor the SBAR guide were in use in government sector hospitals at the time of the trial nor are they currently in use, so the concepts were new to the nurses. The MEWS chart replaced the existing observation chart (Additional file 3) for consented patients for the duration of the 31-day trial. A condition of ethical approval of the study by the Western Cape Department of Health was that hospital staff would not be burdened with the process of obtaining consent from patients. During the trial, two researchers (UK, DB) interviewed patients on 11 occasions on the three intervention wards.

On these occasions, written and verbal explanations of the study were given to eligible patients in any of three languages (English, Afrikaans and isiXhosa). For cultural reasons, some patients requested time for discussion with their families before giving written consent. If patients were illiterate or if they customarily deferred to health professionals, the researchers or research assistants fluent in their language read, translated and explained the consent form. In this way, we took care to ensure that patients understood the implications of their signed consent. Since most patients were unfamiliar with the various forms of nursing documentation, this involved explaining that researchers wanted to see the quality of recordings of their blood pressure for example, and nurses’ responses to any abnormal recordings. Signed consent or a witnessed ink thumb print was taken from every patient participating: no consent by patient consultee was authorised. No identifiable patient data were extracted from patients’ records. The same consenting process for record review was followed for patients in control trial arms on 11 occasions but with the assistance of more research assistants (SG, RR and VvH in addition to UK) because of geographical proximity.

### Data Collection

Data collectors (RR, SG, FB, VvH) were not informed of the study aims and objectives, to reduce observer bias [22]. They were required to treat the data as confidential and undertook in writing not to publish the data or any portion thereof or any documentation relating to the data, other than for the purpose of carrying out their mandate.

Raw data for each patient (vital signs recordings on the MEWS chart and data recorded on the SBAR communication guide) were captured directly on IBM SPSS datasheets (version 22 for Windows, IBM Corp 2011) on the hard drive of a personal computer secured by password-protected access (UK, DB). Baseline patient clinical and demographic characteristics were located on each patient’s personal folder and entered into the SPSS datasheets.

Trial outcomes were assessed by criterion-based clinical record review [2], for example, assistance summoned: yes = 1, no = 0, and did not require subjective interpretation. Vital signs were coded according to MEWS criteria (lower 1, lower 2, lower 3, 0, upper 1, upper 2, and upper 3). These criteria were used to assess the appropriateness of responses (summoning or not summoning assistance). Double data-entry was undertaken by an independent investigator (SJ) on 28 (9.6%) records randomly selected using SPSS. Following discussion on interpretations, two further random samples of 7 and 34 records (total 69/292, 23.6%) were checked [23]. Data, labelled as trial arms A and B to allow analysis to be blinded, were passed to the analyst (SJ). We followed Consolidated standards of reporting trials (CONSORT) guidelines extended to pragmatic RCTs [24] (Additional file 4) and the local University of Cape Town (UCT) (CRC) Standard Operating Procedures (SOPs).

### Data analysis

Demographic and clinical data were described for both arms. Frequency counts and cross-tabulations were used to compare the prevalence of documentation and responses in both arms during the study period. Where numbers were sufficient, associations were explored in bivariate analyses. Pearson’s chi-squared test was used to compare proportions. Where cells contained fewer than the minimum expected count, Fisher’s exact test was substituted. Findings for binary outcomes were expressed in odds ratios with 95% confidence intervals (CIs) [25]. Small numbers of values for outcome variables precluded multivariate analyses [26].

## Results

### Response rate and follow up

**Fig. 1:**
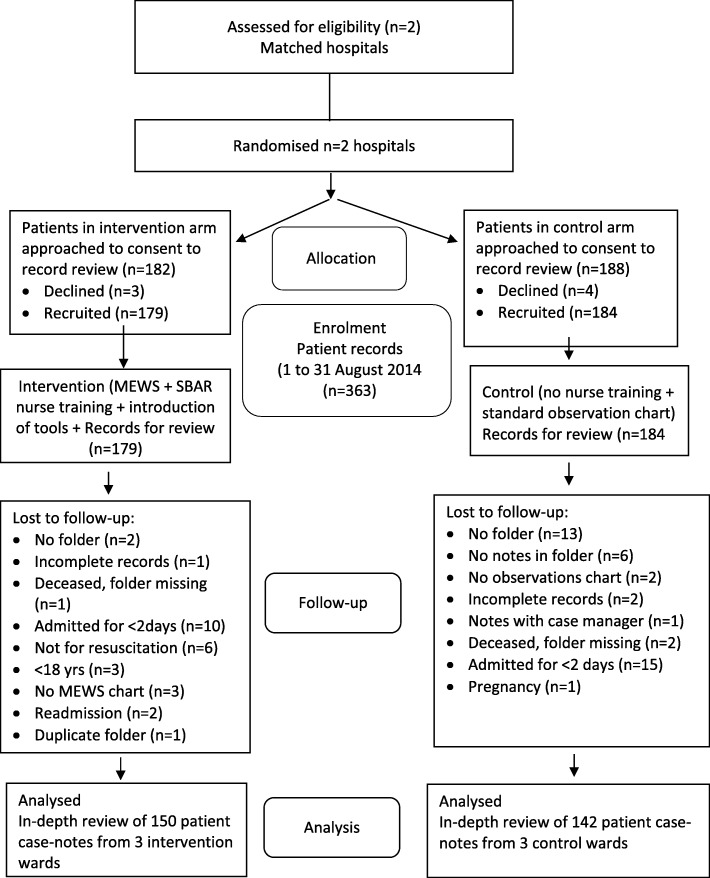
Consolidated standards of reporting trials (CONSORT) flow diagram of criterion-based record review process of the trial

Following provisional assurances, three of the six acute hospitals declined to participate, leaving two matching hospitals and a third that could not be matched. Six wards were purposively recruited (two surgical, one medical), respectively, in the two participating hospitals (three wards in each hospital). The hospitals declined to participate for various reasons (one after an 8-month delay): recent introduction of a pilot electronic hospital record system which included the standard vital signs observations chart (the MEWS was paper-based), “too busy”, and the HREC’s approved patient consenting system by poster was deemed unacceptable. We resorted to individual written patient consent. This effectively excluded patients too ill to be approached and to sign fully informed consent and those most likely to experience SAEs, and precluded recruitment of sufficient patients to achieve the required sample size of 612 patients, with the expected prevalence of SAEs. We obtained written consent from 363 patients for their records to be reviewed: 292 (80.4%) were available for analysis, 150 (51.4%) in the intervention arm (*n* = 101 surgical; *n* = 49 medical) and 142 (48.6%) in the control arm (*n* = 98 surgical; *n* = 44 medical) as shown in the CONSORT flow diagram (Fig. 1). Three participants in the intervention arm had no MEWS chart and were excluded, and a further nine had no total MEWS recorded but were retained.

#### Participant characteristics

Patients were not individually randomised, and we compared patients in intervention and control wards. Demographic details and the prescription of opiates, sedatives, diuretics and anti-hypertensives received on the day of abnormal vital signs recordings are described in Table 1.

**Table 1:** Baseline demographic and clinical characteristics of patients in intervention and control arms

Characteristic	Intervention arm (*n* = 150)	Control arm (*n* = 142)
Male, number (%)	96 (64.0)	55 (38.7)
Age in years:		
Mean [SD]	53.7 [16.2]	49.9 [17.5]
Median [25th to 75th centile]	56 [40.8–65.0]	52 [34.0–62.3]
Full range	19–92	18–92
Length of stay (days):		
Mean [SD]	6.0 [3.8]	8.0 [5.9]
Median [25th to 75th centile]	5.0 [3.0–7.0]	6.5 [4.0–9.3]
Full range	2–21	2–31
Pre-existing co-morbidity	Number (%)	Number (%)
Hypertension	71 (47.3)	48 (33.8)
Type of admission		
Surgical admission	101 (67.3)	92 (64.8)
Medical admission	49 (32.7)	50 (35.2)
GA during hospitalization period	44 (29.1)	36 (25.4)
Prescription medicines and vital signs		
Anti-hypertensives that day^a^	67 (44.4)	41 (28.9)
Diuretics that day^a^	53 (35.1)	30 (21.1)
Opioids that day^a^	104 (68.9)	32 (22.7)
Other sedatives that day^a^	51 (33.8)	13 (9.2)
Vital signs prescribed by doctor^b^	37 (24.7)	44 (31.0)

*SD* standard deviation, *GA* general anaesthetic

^a^The day on which abnormal physiological result was recorded

^b^Type and frequency of observations ordered and documented by a medical doctor

### Recordings

Baseline differences in recordings of the numbers of patients with each of respiratory rate, oxygen saturation, temperature, heart rate, systolic BP, level of consciousness, urine output and with each of the clinical signs of deterioration are presented in Table 2.

**Table 2:** Number of patients with physiological and clinical parameters recorded in the two trial arms

Parameter	Intervention arm *n* = 150 Number (%)	Control arm *n* = 142 Number (%)	OR (95% CI)	Chi-square df = 1	*P* value
Vital signs					
RR recorded	150 (100.0)	141 (99.3)			
Oxygen sats recorded	81 (54.0)	25 (17.6)	5.49 (3.21–9.41)	41.78	< 0.001
HR recorded	150 (100.0)	142 (100.0)			
SBP recorded	150 (100.0)	142 (100.0)			
Temperature recorded	150 (100.0)	142 (100.0)			
Level of consciousness recorded	135 (90.0)	108 (76.1)	2.83 (1.47–5.47)	10.16	0.001
Urine output recorded^a^	41 (27.3)	66 (46.5)	0.43 (0.27–0.71)	11.51	< 0.001
Clinical parameters^b^					
Perfusion recorded	48 (32.0)	42 (29.6)	1.12 (0.68–1.84)	0.20	0.65
Skin colour recorded	58 (38.7)	9 (6.3)	9.32 (4.40–19.74)	43.12	< 0.001
Pain recorded	110 (73.1)	54 (38.0)	4.48 (2.73–7.36)	36.93	< 0.001
Sweating recorded	76 (50.7)	1 (0.7)	144.81 (19.74–1062.35)	93.78	< 0.001
Wound oozing recorded	72 (48.0)	28 (19.7)	3.76 (2.23–6.34)	25.91	< 0.001
Pedal pulses recorded	75 (50.0)	25 (17.6)	4.68 (2.73–8.01)	33.99	< 0.001
Glucose recorded	44 (29.3)	101 (71.1)	0.17 (0.10–0.28)	50.97	< 0.001
Hb recorded	67 (44.7)	89 (62.7)	0.48 (0.30–0.77)	9.51	0.002
Looks unwell recorded	100 (66.7)	4 (2.8)	69 (24.13–197.27)	129.69	< 0.001

No information found taken as sign/symptom not recorded, rather than the patient was not experiencing this sign/symptom. *P* values are reported to two decimal places, except where the third place gives additional information as advised: *P* values >0.01 should be reported to two decimal places, those between 0.01 and 0.001 to three decimal places; *P* values smaller than 0.001 should be reported as *P* < 0.001 (The New England Journal of Medicine, https://www.nejm.org/author-center/new-manuscripts, accessed 30.10.2019) [27]

*RR* respiratory rate, *sats* saturation percentage, *HR* heart rate, *SBP* systolic blood pressure, *Hb* haemoglobin

^a^Urine passed in the toilet was counted as recorded

^b^Clinical parameters were not listed on the standard observation chart (control arm) but data were found in the patients’ progress notes and/or other documents

There was a statistically significant difference in recording between the trial arms for physiological parameters listed on the MEWS observations chart but omitted from the standard ward observations chart: oxygen saturation (81/150, 54.0% vs 25/142, 17.6%, OR 5.49 [3.21–9.41], *P* < 0.001) and level of consciousness (135/150, 90.0% vs 108/142, 76.1%, OR 2.83 [1.47–5.47], *P* = 0.001). All seven vital signs were recorded more often on the MEWS observations chart in the intervention arm than on the standard chart in the control arm and the difference was close to statistical significance (33/150, 22.0% vs 19/142, 13.4%, OR 1.83 [0.98–3.40]).

There was a statistically significant difference between the trial arms for reporting clinical parameters listed on the MEWS but not the standard chart (Table 2). Skin colour (pallor/cyanosis), pain, sweating, wound oozing, pedal pulses and “looks unwell” were more frequently recorded on the MEWS. Glucose and haemoglobin levels were more frequently recorded on standard charts (Table 2).

### Summoning assistance

#### Assistance summoned for a red MEWS warning - score of 3

The primary outcome, nurses’ appropriate reporting behaviour, assessed from documentation in patients’ records, was disappointing in both arms. Assessment of a patient is required immediately when a single physiological parameter falls outside predetermined ranges printed in red on the MEWS chart with a score of 3. The number of patients with 0–4 recordings of level-3 MEWS is shown in Table 3: no patients had more than 4 such recordings. The difference between the two trial arms in the number of patients with any vital signs recordings of level-3 red MEWS was statistically significant (45/150, 30.0% vs 81/142, 57.0%, *P* = < 0.001, OR 0.32 [0.20–0.52]), indicating that the control-arm participants were more likely to have disturbed physiology. Some patients had more than one recording at red level 3. More patients in the control arm had one, two or four such abnormal recordings. Fewer patients with abnormal vital signs at red level 3 in the intervention arm were referred for medical attention, that is, they had assistance summoned (2/45, 4.4% vs 11/81, 13.6%, OR 0.29 [0.06–1.39]) (Table 3).

**Table 3:** Patients with level-3 (red) MEWS vital signs recordings and responses in two trial arms

Number of level-3 MEWS vital signs recordings*	Intervention arm (*n* = 150) Number (%)	Control arm (*n* = 142) Number (%)	OR (95% CI)	*P* value
0	105 (70.0)	61 (43.0)		
1	32 (21.3)	62 (43.7)		
2	12 (8.0)	16 (11.3)		
3	1 (0.7)	1 (0.7)		
4	0	2 (1.4)		
Patients with any red MEWS	45 (30.0)	81 (57.0)	0.32 (0.20–0.52)	< 0.001
Assistance summoned				
Yes	2 (4.4)	11 (13.6)		
No but should have	43 (95.6)	70 (86.4)	0.29 (0.06–1.39)	0.03, 0.02 with Yates’ correction

*MEWS* modified early warning score

*P* value reporting, see “Table 2” notes

^a^We excluded:

• Temperature recordings, due to inconsistent measurement techniques and falsely low readings

• Urine output due to low numbers of recordings and almost zero recording of volume in both arms

#### Physiological parameters with MEWS red (3) and yellow (2) warning scores

While assessment of a patient is required immediately when a single physiological parameter triggers at a score of 3 (red) on the MEWS chart, review of a patient is required 5 min after a single physiological parameter triggers at a MEWS of 2 (yellow). All abnormal recordings of vital signs reaching the trigger thresholds of a MEWS level 2 or 3 for respiratory rate, oxygen saturation, heart rate, systolic BP and level of consciousness for each patient are detailed in Table 4. Some patients had more than one recording at a MEWS level 2 or 3, but this is not indicated in Table 4. The number of recordings of abnormal vital signs that should have triggered at a MEWS level 2 or 3 for these parameters reached 165 in the intervention arm (but did not in 159/165, 96.4%) and 178 in the control arm (but did not in 158/178, 88.8%) (Table 4).

**Table 4:** Number of patients with recordings of abnormal vital signs that did/did not trigger responses at MEWS of 3 or 2 in two trial arms

MEWS	Vital signs	Intervention arm (*n* = 150) Number (% of total)	Control arm (*n* = 142) Number (% of total)	OR (95% CI)	*P* value (from Fisher’s exact test)
	Respiratory rate (RR) (bpm)				
3	25–50	9 (6.0)	61 (42.9)		
2	21–24	34 (22.7)	9 (6.3)		
	Total abnormal RR	43 (28.7)	70 (49.3)		
	Rescued/assistance summoned	Number (% of abnormal)	Number (% of abnormal)		
	Yes, and should have	1 (2.3)	1 (1.4)	1.64 (0.10–26.97)	0.73
	No, but should have	42 (97.7)	69 (98.6)		
	Oxygen saturation (%)				
3	78–91	12 (8.0)	6 (4.2)		
2	92–93	7 (4.7)	3 (2.1)		
	Total abnormal sats	19 (12.7)	9 (6.3)		
	Rescued/assistance summoned	Number (% of abnormal)	Number (% of abnormal)		
	Yes, and should have	0	5 (55.6)	Cannot be computed	0.003
	No, but should have	19 (100.0)	4 (44.4)		
	Heart rate (HR)				
Upper 3	≥ 131	8 (5.3)	7 (4.9)		
Lower 3	≤ 40	1 (0.7)	1 (0.7)		
Upper 2	111–130	35 (23.3)	29 (20.4)		
	Total abnormal HR	44 (29.3)	37 (26.1)		
	*Rescued / assistance summoned*	Number (% of abnormal)	Number (% of abnormal)		
	Yes, and should have	2 (4.8)	1 (2.7)	1.71 (0.15–19.70)	0.66
	No, but should have	42 (95.2)	36 (97.3)		
	Systolic Blood Pressure (SBP)				
Upper 3	≥ 220 mmHg	1 (0.7)	4 (2.8)		
Lower 3	≤ 90 mmHg	28 (18.7)	23 (16.2)		
Lower 2	91–100 mmHg	28 (18.7)	32 (22.5)		
	Total Abnormal SBP	57 (38.0)	59 (41.5)		
	Rescued/assistance summoned	Number (% of abnormal)	Number (% of abnormal)		
	Yes, and should have	1 (1.8)	11 (18.6)	0.08 (0.01–0.63)	0.003
	No, but should have	56 (98.2)	48 (81.4)		
	Level of consciousness (LOC)				
3	Reacting to voice/pain/unresponsive	2 (1.3)	3 (2.1)		
	Total abnormal LOC	2 (1.3)	3 (2.1)		
	Rescued/assistance summoned	Number (% of abnormal)	Number (% of abnormal)		
	Yes, and should have	2 (100.0)	2 (66.7)	Cannot be computed	0.36
	No, but should have	0	1 (33.3)		
	Total abnormal recordings	165	178		

*P* value reporting, see “Table 2” notes

*MEWS* modified early warning score, *bpm* breaths/minute, *sats* saturation percentages

#### Respiratory rate

More patients in the control arm (*n* = 70, 49.3%) needed review of respiratory rate than in the intervention arm (*n* = 43, 28.7%). The difference between trial arms in the number of patients whose abnormal respiratory rates triggered assistance was not statistically significant (intervention arm 1/43, 2.3% vs 1/70, 1.4% in the control arm).

There were 9 of 150 patients in the intervention arm (6.0%) and 61 of 142 patients (42.9%) in the control arm with a rapid respiratory rate (≥ 25 bpm) recorded, meeting the criterion for a single red MEWS of 3, suggesting more acute illness in the control arm. None of these triggered a response in either arm. There were no recordings in either the intervention or control arm of a single red MEWS for a low respiratory rate (≤ 8 bpm).

In the intervention arm, the SBAR tool was used appropriately for one patient with a respiratory rate of 22 breaths per minute (bpm) (MEWS of 2). In the intervention arm most patients with abnormal respirations (34/43, 22.7%) had respiratory rates between 21 and 24 bpm (MEWS of 2). In the control arm most (61/70, 42.9%) abnormal respiratory rates were > 25 bpm. Assistance was summoned for two patients (one in each arm) with rates of 21–24 per minute (yellow MEWS of 2) (Table 4).

#### Oxygen saturation

There was a statistically significant difference between trial arms in the number of patients whose abnormal oxygen saturations triggered assistance (intervention arm 0/19 vs 5/9, 55.6% in the control arm; *P* = 0.003, Fisher’s exact test, Table 4). Patients in the intervention arm were less likely to have assistance summoned. There were 12 of 150 patients (8.0%) in the intervention arm and 6 of 142 patients (4.2%) in the control arm with oxygen saturation levels < 92%, indicating acute illness. Of the five responses in the control arm, three (60.0%) were for a MEWS level 3 and two (40.0%) for a MEWS level 2. The SBAR tool was not used to communicate low saturation levels for 19 patients in the intervention arm: 12 were patients with saturations < 92% (MEWS of 3) and 7 were patients with 92–93% saturations (MEWS of 2) (Table 4).

#### Heart rate

More patients in the intervention arm (*n* = 44, 29.3%) than in the control arm (*n* = 37, 26.1%) required review for an abnormal heart rate (Table 4). In the intervention arm none of the eight recordings in the red MEWS range of 3 for tachycardia at ≥ 131 beats per minute triggered a response but there was one response for MEWS level 2. The single recording of bradycardia at a MEWS level 3 triggered a response. In the control arm only one reported incidence of tachycardia at MEWS level 2 elicited a response.

#### Systolic blood pressure

In the intervention trial arm, 57 patients required review for abnormal systolic blood pressure (Table 4). The lower response rate in the intervention arm (1/57, 1.8% vs 11/59, 18.6%) was statistically significant (*P* = 0.003, OR 0.08 (0.01–0.63)). No low systolic blood pressure (SBP) (≤ 90 mmHg) recordings in the red range triggered a response, and neither did the single recording for high SBP (≥ 220 mmHg). The single response was for MEWS level 2 (92 mmHg) and the SBAR tool was used appropriately.

In the control arm 23 of 142 (16.2%) patients had a red MEWS value recorded for low SBP (≤ 90 mmHg), and 4 of 23 (17.4%) were actioned comprising 4/11 (36.4%) of the total responses. There were 4 of 11 (36.4%) responses for recordings of high SBP (≥ 220 mmHg) and 3 of 11 (27.3%) responses from 32 recordings for MEWS level 2 (91–100 mmHg).

#### Level of consciousness

There were few reported incidents: in the intervention arm both reported incidents of a disturbed level of consciousness were actioned, as were two of three reports (66.7%) in the control arm.

#### Temperature

At a critical MEWS level of 3, there were 65/150 patients (43.6%) in the intervention arm with abnormally low temperature recordings (≤ 35 °C) as their only abnormal sign; none of these elicited a response. Response rate was similarly low in the control arm. Ward digital thermometers were not calibrated regularly and the procedure for taking axillary temperatures was questionable: nurses were observed placing the digital thermometer between layers of clothing.

We regarded recordings of temperature readings as unreliable and omitted these from Table 4. Had these recordings been accurate, recordings would have triggered calls for assistance for 45 of 150 (30.0%) patients in the intervention arm and for 81 of 142 (57.0%) patients in the control arm.

#### Urine output

For many patients in both trial arms, no urine output was recorded (intervention arm: *n* = 107, 71.3%; control arm: *n* = 69, 48.6%) and no responses were recorded. In both trial arms a fluid balance (intake/output) chart for recording urine output was used, and this was completed in preference to the MEWS chart in the intervention arm. Most were recorded as passed urine in toilet (PUIT). Volume was seldom measured on MEWS or fluid balance charts (5/142, 3.5% in the control arm and 13/150, 8.7% in the intervention arm).

### Serious adverse events (SAEs)

Few SAEs were recorded and there were no statistically significant differences between trial arms (Table 5).

**Table 5:** Serious adverse events recorded

SAE	Intervention arm *n* = 150 Number (%)	Control arm *n* = 142 Number (%)	OR (95%CI)	*P* value
Any SAE	5 (3.3)	3 (2.1)	1.61 (0.38–6.86)	0.72 (Fisher’s)
Death	*4 (2.7)	1 (0.7)	3.89 (0.43–35.23)	0.37 (Fisher’s)
Prolonged hospitalization	0	2 (1.4)	Cannot be computed	
ICU admission	1 (0.7)	0	Cannot be computed	

*A specialist physician (UK) advised that a young patient diagnosed with chicken pox pneumonia and transferred from the intensive care unit to a medical intervention ward, and whose vital signs were monitored by the standard chart, had died 2 h later. The physician expressed his opinion that had the MEWS been used, the patient’s vital signs would probably have been better monitored, leading to interventions that might have saved his life

## Discussion

It is reported that delayed recognition of deterioration of patients on general wards can be attributed to human-related monitoring failures [28]. Early detection of at-risk patients requires regular and systematic assessment [29]. The primary outcome, nurses’ responses to early signs of clinical and physiological deterioration, was disappointing in both arms and the SBAR communication guide for reporting clinical deterioration in intervention wards was rarely used. Importantly, and for the first time, this trial offers limited support for the assumed superiority of EWS/MEWS. This “no benefit” conclusion can only be extended to the same or similar settings and conditions pertaining to this trial: evaluation of paper-based EWS instruments not previously used routinely, gatekeepers declining access to researchers for ethically approved studies, and the requirement for patient consent for testing new nursing documentation posing low/no risk to patient care. No similar studies were identified.

Nurses in the control arm were more likely to respond to abnormal physiology, except for level of consciousness. Other than (likely spurious) low temperature recordings, no assistance was summoned for a single red MEWS for critical illness for 96% of affected patients in the intervention arm and 86% of patients in the control arm. Nurses largely relied on SBP, oxygen saturation and level of consciousness when deciding on whether to summon assistance, and tended to disregard respiration rate. Nurses’ measurement of respiratory rate was found to be unreliable [30].

Although axillary temperature is not considered accurate [31] and is about 1 °F lower than oral temperature [32], it was the conventional method of measurement on public sector hospital wards in Cape Town at the time. It is unusual for nurses to report low temperature recordings.

There was a statistically significant difference between the trial arms for recording of physiological parameters listed on the MEWS observations chart but not on the standard ward observations chart: oxygen saturation (OR 5.49 (3.21–9.41), *P* < 0.001) and level of consciousness (OR 2.83 (1.47–5.47), *P* = 0.001) (Table 2).

As reported elsewhere [1] clinical signs such as skin tone, sweating, nausea or nurses’ intuitive assessment of the patient being “just not right” and “looking unwell” [33] should be monitored regularly to limit avoidable, serious adverse events (SAEs) such as cardiac arrest, urgent and unanticipated admission to an intensive care unit (ICU) or even death. There was a statistically significant difference between the trial arms for clinical parameters listed on the MEWS observations chart but not on the standard ward observations chart: skin colour (OR 9.32 (4.40–19.74), *P* < 0.001), pain (OR 4.48 (2.73–7.36), *P* < 0.001), sweating (OR 144.81 (19.74–1062.35), *P* < 0.001), wound oozing (OR 3.76 (2.23–6.34), *P* < 0.001), pedal pulses (OR 4.68 (2.73–8.01), *P* < 0.001), and looks unwell (OR 69.0 (24.13–197.27), *P* < 0.001). The MEWS ensured that such measurements were more comprehensive, with two exceptions for glucose (OR 0.17 (0.10–0.28), *P* < 0.001), and haemoglobin (OR 0.48 (0.30–0.77), *P* <0.002) levels. There were too few SAEs to draw any conclusions and the higher numbers of SAEs in the intervention arm, despite fewer abnormal vital signs, may have been due to complexities and intra-hospital factors other than the type of observation chart.

### Limitations, challenges and strengths

Our pilot study was not delivered as intended. However, this trial has raised important questions about ethical and regulatory challenges related to PCTs [16], including the role of gatekeepers [17], and determination of what constitutes “no more than minimal risk” research [18].

#### Gatekeeping recruitment and delivery

This was a two-hospital comparison, where, for administrative and practical considerations, and to avoid contamination [34], trial arm was dictated by hospital. Whilst we acknowledge the limitation of this design [35], this was the only available option after three hospitals declined involvement. With only one hospital in each arm, we were unable to adjust for hospital characteristics, overt and tacit, and we acknowledge this limitation. Preliminary data had indicated that patients were similar in the two participating hospitals, and there were no marked dissimilarities between the recruited samples. However, on data collection, consenting patients in the intervention hospital were slightly older and more likely to have been medicated. Similarly, recordings of vital signs (unavailable before the study) indicated that the patients in the control arm were probably sicker (Tables 1, 2).

Gatekeepers critically shaped our trial [17]. During recruitment, we were denied access to a hospital after an 8-month delay, justified by the commencement of a pilot study on an electronic document system that included the standard vital signs observations chart. The duration of the training programme was reduced by hospital managers, from a planned 8 h to 2 h, and this fell to 30–60 min on night duty, due to staff shortages. We do not know the number of relief, agency or part-time nurses who completed the MEWS charts without any training from full-time staff. Failure to train agency nurses may have affected study findings: nurse managers did not give these nurses the opportunity to receive the training on the intervention, because they were not considered to be the hospital’s stable workforce. The clinical impact of professional training courses varies [36]: whilst some authors report the success of 1-day multi-media multi-professional training courses [37, 38], others report no significant changes [39]. Our teaching was limited to nurses in full-time employment and the strategy included a didactic approach, followed by clinical scenarios to guide filling in blank MEWS charts, but might have been enhanced by simulation [13]. No simulation laboratory was available in the hospital, and, at the time, there were no facilities for online teaching and learning [13].

#### “No more than minimal risk” research

The study was less representative and smaller than planned. Patient records could not be randomly selected as the individual patient consenting process effectively excluded ill patients and proved to be a barrier to recruiting sufficient patients to achieve the required sample size. We had aimed to recruit 51 patients in each of 12 wards across 6 hospitals. We were able to recruit 292 eligible patients across 6 wards in 2 hospitals. We recognise that this lower effective sample size detracts from our trial and subsequent decision-making. Although the previous Research Ethics Committee (REC) in 2009 and funders (National Research Foundation, South Africa) had agreed that consent would be obtained at cluster (ward) level, restrictions were imposed by the REC in 2014 on the consenting process for collection of anonymised data from hospital records [40].

As this was a trial of nursing documentation, without patient contact, and patients are not normally consulted about the design of nursing charts for recording vital signs, or other nursing documentation, we had anticipated compliance with the Ottawa Statement, integral to the CONSORT statement [41–43] and recommendations for PCTs [16, 18]. The limited available resources to consent patients are reflected in the reduced number of participants, indicating that findings must be interpreted with caution and regarded as preliminary. Largely due to the exclusion of the patients who were the most ill, there were small numbers in outcome variables (two subjects in the key outcome in the intervention arm), precluding adjusted analyses [26]. We made no adjustment for multiple testing. A larger sample would have been unlikely to reverse our findings; however, we can only speculate as to whether the original multi-site study with an unbiased patient sample would have yielded similar findings.

HIV status was not a consideration for this study. Zambia, a developing country, with a significant HIV burden, demonstrated no significant difference in early warning scores when comparing patients with different HIV status (HIV positive, negative or unknown), *P* = 0.51 [44].

#### Generalisation

From single-centre research, we cannot assume that findings can necessarily be generalised to settings where the prevalence of the conditions under consideration or nurse-to-patient ratio or nurses’ educational levels or communication ethos may differ [45, 46]. Furthermore, resources did not extend to qualitative analyses of nurses’ perceptions of the MEWS charts [47]. However, the staffing and case mix are representative of hospitals in Cape Town. The non-participation of three hospitals and patients unwilling or too ill to consent to the review of records by researchers introduces a potential for volunteer bias. We cannot generalise our findings to sicker patients or settings less willing to participate in research [48], and clinical trial volunteers are often of higher socioeconomic status than those who decline [49]. Had all patients been included, the prevalence of the “summoning assistance” outcome variables might have been sufficient for a more robust analysis.

#### Sources of bias

Blinding: it is rarely possible to blind staff participants in trials of documentation, but patients were unlikely to have been aware of their allocation. As the MEWS charts were in patients’ records in the intervention wards, it was not possible to blind the independent record reviewers to trial arm. Blinding of staff during administration of the intervention, data capture and outcome assessment in non-pharmacological [40], cluster [50] or pragmatic trials may be impossible [24], increasing the risk of bias [22] in line with observers’ expectations [51]. Bias in the detection of outcomes was minimised, but not removed, by a pre-arranged data collection template and the low subjectivity of outcome data extracted from standardised documentation, such as vital signs’ records [35]. There were no obvious differences between the two arms in outcomes; therefore data analysts could not guess whether A or B was the intervention arm. Findings favouring the control arm offer no evidence that the study team were influenced by the Rosenthal effect [51] and entrapment by prior expectation [52]. Systematic review indicates that some 3% unblinded assessments are likely to be misclassified [53], which would not materially affect interpretation of our study.

Our findings are dependent on the reliability and completeness of patient records. There were several instances where summoning senior assistance would have been warranted, but this was not documented: we could only conclude that this was not done [54]. We have no reason to suppose that this and the Hawthorne effect [55] would have affected the arms unequally.

## Conclusions

Despite shortcomings, our pilot trial can contribute to a “learning healthcare system” [56] in which continuous learning takes place in the context of routine patient care towards providing answers to clinical questions [16]. The revised CT MEWS observations chart improved recording of certain physiological and all clinical parameters when compared to the standard chart but did not improve nurses’ ability to identify early signs of clinical deterioration and summon assistance. Recruitment of only two hospitals limits generalisation of these findings. Further work is needed on educational preparation for the CT MEWS/SBAR and testing impact on nurses’ reporting behaviour.

## Supplementary information

**Additional file 1.** The Revised Cape Town MEWS vital signs observations chart.

**Additional file 2.** The SBAR communication guide.

**Additional file 3.** Existing Department of Health observation chart.

**Additional file 4.** Checklist: CONSORT statement extended to pragmatic trials.

## Data Availability

The datasets used and/or analysed during the current study are available from the corresponding author on reasonable request.

## Abbreviations

CRC: Clinical Research Centre
CT: Cape Town
DOH: Department of Health
EWS: Early warning score
HREC: Human Research Ethics Committee
LOC: Level of consciousness
MET: Medical Emergency Team
MEWS: Modified Early Warning Score
NEWS: National early warning score
NHI: National Health Insurance
PACTR: Pan African Clinical Trials Registry
PCT: Pragmatic clinical trial
RPN: Registered Professional Nurse
SAE: Serious adverse event
SBAR: Situation-background-assessment-recommendation
SOPS: Standard operating procedures
UCT: University of Cape Town
