# Supplementary material for: Testing effectiveness of the revised Cape Town modified early warning and SBAR systems: a pilot pragmatic parallel group randomised controlled trial
Source: Trials. 2019 Dec 30;20:809. doi: 10.1186/s13063-019-3916-0 (PMC6937946; doi:10.1186/s13063-019-3916-0)
Supplement: Supplementary file 2 — Additional file 2. The SBAR communication guide. [file 13063_2019_3916_MOESM2_ESM.pdf]

Patient label

SBAR Communication document:

Instructions:

Please obtain a complete set of vital signs.
Complete the SBAR communication document quickly before calling the doctor by filling in:
the required information or using tick box ✔ (Yes) **X** (NO) or **ND** (Not done).
Keep your descriptions brief and relevant to why you are calling.
Ensure you have the patient's 'OBS' chart and medication charts at hand when calling the doctor.
Be prepared for a second witness if medications are ordered.

Time Dr. alerted:___________________ Time DR. responded _______________ Date: ______________________

**S**<br>Situation	This is ______________________________ calling from ______________________________ (State your name, title and location).<br>I am calling about patient ____(State patients name).<br>The problem I am calling about is ____<br>____<br>____<br>____<br>(Provide disturbed vital signs, **OR** the reason why you are concerned about the patient).<br>The patient's resuscitation status is 'for resuscitation' ☐ or 'not for resuscitation' ☐
**B**<br>Background	The patient was admitted on ____(Admission date and time if known)<br>Admission diagnosis is ____<br>A **brief** relevant history for this patient is ____<br>____<br>____<br>(Provide current weight **and** a quick summary of any secondary diagnosis such as diabetes, hypertension **as well as** procedures/ operations / tests related to the current problem **and** if the patient has any allergies).<br>Current treatment includes ____<br>____<br>(Provide a **brief** summary of current treatment such as intravenous access, intravenous fluids given, medications recently given or of importance, oxygen therapy and oral intake).<br>This is a change from ____<br>____ (Describe briefly what the previous condition was).
**A**<br>Assessment	**Current vital signs are**: Respiratory rate ______ Oxygen saturation %________ On oxygen %/L/min __________ **or** Room air ☐<br>Temperature ____________ Heart rate ____________ Blood pressure ___________/____________<br>Glasgow coma scale (_____/15) **or** Alert ☐ Responds to Verbal ☐/Pain ☐ is Unresponsive ☐<br>**ONLY IF APPLICABLE complete and state the following:**<br>**Skin colour**: Pale ☐ Cyanosis ☐ **Periphery**: Warm (Capillary refill time <2 seconds) ☐ or Cool (CRT>2 seconds) ☐<br>**Pupils**: Equal ☐/Pinpoint ☐ Normal size ☐ Dilated ☐/Reacting to light ☐/**Mood**: ☐ Lethargic ☐ Confused ☐ Agitated ☐<br>The patient is **complaining** of ____<br>____<br>**Pain experienced**: No pain ☐ Mild pain ☐ Moderate pain ☐ Severe pain ☐<br>**Sweating**: ☐/**Wound ooze**: ☐/**Pedal pulses**: Normal ☐ Weak ☐ or Absent / ☐ **Blood glucose**: _______ /**Finger prick Hb**:_______<br>Additional item: Urine output has decreased over the last few hours ☐
**R**<br>Recommendation	I would like you to see the patient now ☐ in the next 30 minutes ☐<br>Is there anything you would like me to do in the meantime? ____<br>____<br>____<br>(**If medications are ordered**): While I have you on the phone may I get a second witness? ☐<br>(**If not coming to see the patient now**): Do you want to be notified for any reason? ______________________________<br>____<br>If no improvement, when should I call again? ____

Compiled by Debora Whitaker R/N UCT Division of Nursing and Midwifery (Supervisor Dr. Una Kyriacos, UCT/ Co-Supervisor Dr. Sue Jordan, Swansea University) based on SBAR report to a physician, Magee-Women's Hospital of UPMC © 2008 (Woodhall et al. 2008).
