# Supplementary material for: Testing effectiveness of the revised Cape Town modified early warning and SBAR systems: a pilot pragmatic parallel group randomised controlled trial
Source: Trials. 2019 Dec 30;20:809. doi: 10.1186/s13063-019-3916-0 (PMC6937946; doi:10.1186/s13063-019-3916-0)
Supplement: Supplementary file 3 — Additional file 3. Existing Department of Health observation chart. [file 13063_2019_3916_MOESM3_ESM.pdf]

WESTERN CAPE GOVERNMENT HEALTH

STANDARD OBSERVATION FLOW CHART

NAME	
FILE NUMBER	WARD
SERVICE	

DAY										
DATE										
TIME										

TEMPRATURE C°

40

39

38

37

36

BLOOD PRESSURE
HEART RATE
RESPIRATION

200
190
180
170
160
150
140
130
120
110
100
90
80
70
60
50
40
30
20
10

WEIGHT											
STOOLS											
DRAINAGE											
HGT gm%											
Hb mmoL/L											
URINE	LEUCCYTES										
	NITRTES										
	BLOOD										
	PROTEIN										
	GLUCOSE										
	KETONES										
	UROBILINOGEN										
	BILIRUBIN										
	pH										
	SG										
